# Constructing inclusive teacher identity in a Finnish international teacher education programme: Indonesian teachers' learning and post-graduation experiences

**DOI:** 10.1016/j.heliyon.2023.e16455

**Published:** 2023-05-19

**Authors:** Satia Zen, Eero Ropo, Päivi Kupila

**Affiliations:** Faculty of Education and Culture, Tampere University, Finland

**Keywords:** Inclusive education, Teacher Identity, International teacher programme, Identity, Construction, Positioning

## Abstract

This article explores how Indonesian teachers continuously reconstruct their identity as inclusive teachers during learning and after graduating from a Finnish master's degree Programme. Taking a narrative and dialogical perspective on identity construction, we analyse the teachers' narratives about their experiences during learning and after graduation when they returned to their respective schools. The findings illustrate the process of teacher identity construction to be inclusive teachers as a repositioning loop. Furthermore, the dynamics of self and others' repositioning provide insights into challenges and contradictions in implementing inclusive education in the post-conflict and post disaster context. The study may provide insights into how teacher identity construction influences inclusive education implementation and its potential to describe the dynamics of this process.

## Highlights

• International Teacher Programme promote construction of inclusive teacher identity.
• Identity construction to be inclusive teachers continues after graduation.
• Dynamics of this process can be described as a repositioning loop.
• Contradictory positions reflect different inclusive education implementations.

## 1 Introduction

**Fig. 1:**
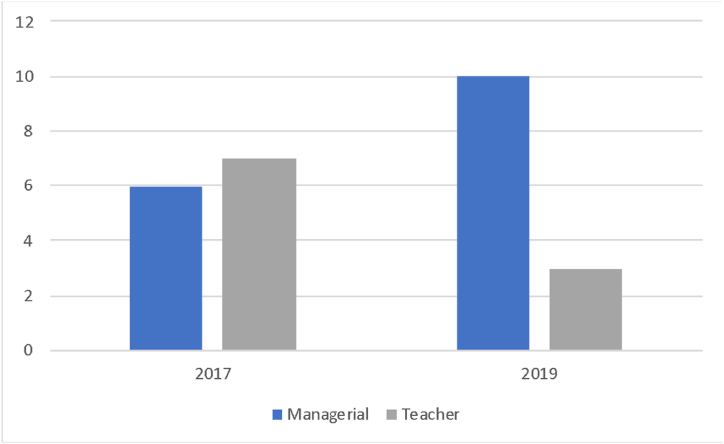
Participants' roles in 2017 and 2019.

This study aims to explore and describe teacher identity construction, specifically the process of becoming more inclusive. This process began during participation in an international teacher programme and continued after graduation. The participants were teachers from Indonesia studying on the International Master's Degree Programme organised by a Finnish university. Specifically, the study focuses on how participants make sense of inclusivity and diversity in their narratives of experiences during these periods. The study is part of a larger research project that explores the dynamic processes of teacher identity reconstruction through positional negotiation of Indonesian teachers participating in Finnish international teacher education programmes (see Refs. [43,44]) (see Fig. 1).

Conceptually, the study is based on a dialogical perspective of teacher identity reflecting the dynamics of their positioning in differing contexts [[2,22,44]]. The study reports how participants reconstructed their identities narratively through diverse positionings during their studies and after graduation. This positioning may come from participants' past and present experiences, originating in social and personal contexts that may continue or discontinue at some point of their narrative [3]. The narratives reflect participants’ negotiation processes as a result of exposure to inclusive education in the Finnish context and describe the dialogical processes of becoming more inclusive.

Definitions of the inclusive education movement have been changing across national and sociocultural boundaries, shaping policies in teacher education and the development of an inclusive education agenda in a particular context [42]. From this perspective, the International Teacher Programme may enable teachers to expand the concept of inclusive education [7]. Teachers studying on the international programme experienced and observed two different implementations of inclusive education. Furthermore, the participants who were facing different ways of being a teacher may have challenged their pre-existing beliefs and values [40]. The exposure challenges participants’ pre-existing beliefs and understanding about inclusive education which may have been part of a belief system and connected to their sense of professional identity [16]. Hence, studying on the International Teacher Programme not only changes their understanding of inclusive education but also stimulates teacher identity reconstruction.

The process of identity construction to be inclusive teachers in this study is framed as an internal negotiation process between two different contexts of inclusive education through positioning and repositioning. Therefore, to explore this negotiation process the study seeks answers to the following questions:

1. In relation to becoming more inclusive teachers, what kind of positional changes participants describe in their narratives?
2. How do the participants describe their positioning during the implementation of inclusive education after graduation in their schools?
3. After graduation, how do participants position themselves in relation to the challenges of implementing inclusive education in a post-conflict and post-disaster context?

## 2 Conceptual framework

### 2.1 Inclusive education approach in international teacher education

The goal of other international teacher programmes in previous studies usually entailed improving teachers' competences to respond to a more diverse classroom, specifically to cultural diversity [15]. The process begins when participants themselves experience diversity and being in the minority, where participating teachers may experience heightened awareness and empathy for future students [40]. An earlier study on this particular programme before its beginning indicates participants' anticipation of changing their identity as individuals and teachers due to being ‘cultural others’ during the programme [35].

However, Olmedo & Harbon [28] propose an important distinction between multicultural education and international education in teacher education. They describe a multicultural education approach preparing teachers to ensure schools and curriculum reflect diversity of the ethnicity, race and culture of the population. The teachers also strive to provide equal opportunities through structural change for those previously underserved groups. Meanwhile, international teacher education is based on a wider global citizenry perspective. This approach aims to prepare teachers by broadening their knowledge base, including awareness of diverse issues that influence the community where they teach and how diverse global issues influence local context [28]. In this study, this approach has the potential to broaden teachers’ understanding about inclusivity that may be relevant considering the context in which the participants of this study are working. The schools where they work are in Aceh province, a region with a history of a post-conflict and post-disaster context that posed complex challenges to its education system.

Based on this perspective, the international teacher education programme provides opportunities for teachers to acknowledge, experience and reflect on the diversity of human conditions and use this understanding to inform their pedagogies. This is in line with one of the most cited influences of these programmes on teaching described as enlargement of reference ([26,41]). This perspective can be applied to see the impact of an international teacher programme in changing teachers’ understanding of diversity and inclusivity. Furthermore, this new understanding may form a core belief about teaching and learning through the identity reconstruction process as inclusive teachers.

In this study, we explore this teacher identity reconstruction process by analysing participants' narratives about diversity and inclusivity. Specifically, the way their languages change to normalise and accept diversity then articulate their practices reflecting inclusive pedagogy, reflecting an ‘inclusive turn’ [1]. For this purpose, we conceptualised changes in the participants' narratives as reflecting a shift, from a ‘categorical and segregative way of looking and managing diversity’ toward ‘creating opportunities to learn for diverse learners’ [9]. These narratives also reflect a dialogical process of negotiation between existing and new positioning associated with the shift. Patterns of repositioning may highlight specific dynamics of teacher identity constructions to be inclusive teachers in this programme.

### 2.2 Teacher identity construction as a narrative and dialogical process

This study views teacher professional identity as dialogically negotiated and narratively constructed [11]. In this perspective, teacher identity is dynamic and multi-faceted, influenced by internal and external factors that are continuously negotiated through a dialogical process ([3,8]). Identity is thus constructed through constant positioning externally through a social position and internally through a personal position [23]. When the participants started to study in the ITP, they were exposed to new positions that may reflect different educational contexts, hence increasing the diversity of their positions and leading to more complex organisations [24].

Concurrently, narrative may be seen as a mediating process that supports identity construction [32]. In their narratives, positioning is one of the ways for individuals to project their identities [18]. In this article the concept of position is understood as a statement in which the interviewee expresses, for instance, his/her relation to certain phenomena or roles in pedagogical activities. The way participants’ narrative describes changes in their positions is viewed as repositioning and indicates negotiation process.

This study elucidates the teacher identity reconstruction process in this programme by exploring the internal negotiation process between positions associated with differing inclusive education implementations described in participants' narratives. Participants' narratives provide insights into a negotiation between diverse positions used to construct meaning about inclusive education through a consonance or dissonance dialogue [24]. These dynamics occur because position represents ‘a speaking personality bringing forward a specific viewpoint and story and driven by its own intentions’ ([3], p. 311) that influences the associated beliefs about duties and rights [21]. Therefore, this process is also associated with ‘differences, tensions, oppositions, and contradictions’ ([24], p. 9).

At the same time, when teachers narrate their experiences in ITP, they are also creating narrative meaning through the process of emplotment, which organises their experiences into ‘temporally meaningful episodes’ ([30], p. 1). Some of these episodes, described as the narrative turning point, may also describe deviation from canonical narratives [13] and serve as ‘a device that further distinguished what is ordinary and expected from that which is idiosyncratic and quintessentially agentive’ ([13], p. 32).

In this study, the narrative turning point represents a departure from participants' previous understanding of inclusive education. The segments describe participants’ repositioning associated with inclusive education implementation in two different contexts, reflecting consonance and dissonance dynamics. In the next section, we will briefly describe how inclusive education is implemented in Finland and Indonesia to provide background and local contexts negotiated in their identity construction.

### 2.3 Inclusive education implementation in Finland and Indonesia

Equality and equity are two concepts that may contribute to the way inclusive education is implemented in Finland. Graham & Jahnukainen [20] describe the focus of the Finnish education system on early detection and normalising ‘special education’ as part of a support system that will not stigmatise students. Teachers can focus on pedagogically responding to students' needs without worrying about schools' performance in external standardised testing. Inclusivity in the classroom therefore starts with teachers accepting students' conditions and providing an optimal environment in which to learn. This approach may suggest that inclusivity is viewed as a core dimension in the learning process that orients teachers' practice and pedagogy to provide opportunities for diverse learners to participate in the learning community [9].

Among the courses on this programme, participants learned about the Finnish approach to inclusive education and observed schools in Finland. They encountered how integrated and early intervention for students' diverse needs in regular school support inclusivity. Lakkala [25] describes this intervention as ‘meaningful participation in learning activities, social acceptance and sense of belonging in a group’ (p. 23). Participants' learning experiences stimulated awareness of different definitions of inclusivity and diversity that lead to different pedagogy, arrangements and practices in Finland. The narratives about comparison and contrast are featured in their narratives.

Inclusive education in Indonesia started to become part of formal schools through Government Decree No. 70 of 2009 on Inclusive Education . In this decree, students with special needs and exceptional abilities are accepted in regular schools, designated as ‘inclusive schools’ and allocated additional funding [29]. This policy exemplifies the ‘integration mode’ of inclusive education into regular schooling [37], where segregation and categories are used to manage diversity in students and viewing disability from an individualistic model [9,37]. Andriana & Evans [6], having studied one of these ‘Inclusive Schools’ in Indonesia, found that ‘inclusive students’ in this school viewed the term ‘inclusion’ as negative and limiting. Additionally, teachers' understanding of this term limits their view of these students as a separate group rather than indication of a diverse school community, thereby contradicting the goal of inclusive education [6].

Other studies on inclusive education in Indonesia also describe some challenges due to how inclusivity is defined and interpreted in practice. Suharto et al. [39] describe the changing terminology of inclusive education for the past three decades to its recent local term, ‘difable’ (different abilities) do not substantially change the consequences for students categorized as ‘difable’, specifically in changing the learning process. This terminology change may reflect the underlying assumption of different abilities as deficient, especially when students' performance through standardised testing is a part of the accountability system for schools in this context. Meanwhile, Sheehy, Budiyanto, Kaye and Rofiah [36] in their research on Indonesian teachers indicate that epistemological beliefs are a stronger predictor of their beliefs in inclusive education and inclusive pedagogy than the type of school where they teach and their experiences. These studies describe the importance of teachers' understanding and beliefs about inclusive education that influence its implementation in Indonesia.

These brief descriptions of inclusive education in the Finnish and Indonesian contexts represent different conceptualisations of diversity and inclusivity. They are then reflected in participants’ positioning in relation to inclusive education. However, learning in ITP provides opportunities for participants to experience and make sense of these differences, reconstruct their identity, and possibly initiate changes on returning to their own school.

## 3 Methodology

The present study is part of a larger qualitative research using a narrative approach to positional changes in participants' narratives and how these positions indicate changes in their identity as teachers by comparing participants' narratives from 2017 to 2019 (see Ref. [43]; [44]). A narrative approach is adopted to design the data gathering and analysis process. The narrative interview is used in the process of gathering participants' narratives, focusing on themes relevant to their experiences during learning and after graduation. Thereafter thematic narrative analysis and positional analysis are used for the analysis process. In this article, we focus on analysing how participants’ understanding of inclusive education changes as part of their identity reconstruction in their narratives about learning (2016–2017) and after graduation (2017–2019). The analysis is focused on their positioning, revolving around themes of diversity, inclusivity and inclusive education. These positionings may also be associated with specific practices in the Finnish and Indonesian contexts, as also with their previous and present practices. At times, the positioning may also indicate future practices.

### 3.1 Context and participants of the study

The context for this study is an international teacher programme conducted in Aceh province, Indonesia, funded by the Sukma Foundation and organised by Tampere University, Finland. From December 2015 until April 2017, the programme was conducted in Sukma Bangsa Schools in Bireuen city, Aceh province, Indonesia, and at Tampere University in Tampere, Finland. The curriculum was mainly based on the International Teacher Master's Degree programme implemented at Tampere University.

The respondents for this study were 13 teachers who agreed to be interviewed for the first time in 2017 and the second time in 2019. These respondents were selected on the basis of their willingness to participate in this study during these two periods. Invitations to participate voluntarily in this study were issued to participants and interviews were scheduled in accordance with their availability. In the first interview, not all those participating in the programme agreed to be respondents in the study. Their educational backgrounds are varied, reflecting diverse routes to becoming teachers in the Indonesian context. Six of the participants graduated from the Faculty of Education and seven graduated from a subject-based faculty. The duration of their teaching experience prior to embarking on the programme also varied, ranging from less than two years (3 participants), two to five years (seven participants) and five to ten years (2 participants).

Prior to participating in an international teacher programme, some participants may have had limited opportunities to study inclusive education. However, due to their working experience in post-conflict and post-disaster contexts, they encounter a diverse range of students' needs and present opportunities for the implementation of inclusive education. The participants taught at schools in three different locations in Aceh province. Participants also taught at different levels (Primary, Middle and Secondary schools). Some participants had additional managerial roles, serving as principals, vice-principals, school counsellors and heads of dormitories, which still included teaching duties. These roles are based on the organisational structure in the school. The following figures describe participants’ roles before studying and during their studies in 2017 and two years after graduating in 2019 (see Fig. 1).

### 3.2 Data gathering

Using narrative interview, we invited participants to describe their experiences while studying and after graduation from the ITP. This interview was deemed to be collaborative in nature and participants were encouraged to use their own words and expand on their stories [33]. The narratives were gathered through interviews conducted on two different occasions by the first author. On both occasions, the interviewer was no longer working on the programme or in the school. The first interview was conducted in the last month of the programme, in April 2017, while the participants were still in Q, Finland. The second one was conducted in July 2019 in Aceh province, Indonesia. The interviews were conducted using the Bahasa Indonesia language and lasted between 40 min and 115 min. The participants’ diverse responses determined the duration of the interviews according to how they expanded their narratives. The narratives from the interviews were then transcribed.

### 3.3 Data analysis

The analysis was conducted using thematic narrative analysis ([31,33]). We organised narratives from the two interviews into two temporal phases lasting for 41 months. The first phase consists of narratives about admission, learning in the programme, research, visiting Finland and graduation (December 2015–April 2017, 17 months). The second phase consists of narratives about their experience of returning to school in July 2017 until June 2019, when they were interviewed for the second time (24 months).

First, we read through each transcription from 2017 to 2019 to see how issues of inclusivity and diversity appeared in participants' narratives. Second, we noted particular segments that might indicate ‘narrative turning points’ concerning these concepts. These segments might be in the form of comparative statements related to what participants knew and did as teachers. There might be statements describing ‘before and after’ and differences between ‘Indonesia and Finland’. Some of these statements might describe a qualitatively different understanding of concepts and practices in teaching and learning, such as assessment, teaching strategy, learning needs, student engagement and others. Third, we analyzed how participants positioned themselves and others in these segments; we considered in what manner the positioning was relational and reciprocal [17]. We identified the ways in which participants described others in their narratives and how they described their own positioning. We then analyzed the dynamics of these positioning in participants' narratives about during learning in ITP and after graduation when returning to their school. The following quotes may describe how the analysis process is conducted in this study.

**Table 1:** Example of analysis process.

Episode	Sample statement	Positional analysis
Learning in the Programme	‘Finnish educators explain about students’ learning needs, I think I never considered my students learning needs too much, I am more concerned about how to find a strategy to deliver my lesson’	Positioning others (Finnish teacher educators) as considering students' needs. Positioning self as a teacher concerned with delivery strategy. New understanding of teaching and learning based on students' needs rather than teaching methods.

The example statements in Table 1 describe the process of analysis by identifying positioning of self, others and accompanying expression of beliefs, thoughts and actions associated with these positions. The statements consist of comparisons between self (the participants) and others (Finnish educators). The theme revolves around a new understanding of identifying learning needs and what teachers should do. Throughout their narratives, the emerging theme of inclusivity with the associated positionings surrounding the theme reflects changes in what they understand about diversity and their beliefs about inclusive education. The narratives also indicate changes in their understanding of being teachers.

The data gathering and analysis processes were supported by the authors’ background and involvement in the programme. The first author is a teacher and school leader with over ten years of experience in Indonesia and was acquainted with most participants before the study. She was also the programme co-ordinator in Indonesia from December 2016 until July 2017. Her proficiency in the Bahasa Indonesia and her understanding of the Indonesian education context contributed to the contextual understanding of the study. The second and the third authors were both teacher educators on the programme. Additionally, the second author was also the programme leader.

The ethical consideration for the whole study is adhere to Finnish National Research Integrity in Human Sciences (TENK, 2019). Based on this guideline, since the study is not involving minors and is not mandatory, the ethical review of the university ethical board is not necessary. However, the university ethical rules required informed consent from all participants of the study listing the risks and rights of participants and clearly stating the voluntary nature of the participation. Therefore, the study is following the ethical guidelines and gathering informed consents for both interview occasions.

## 4 Findings

This study explores how teacher identity reconstruction as inclusive teachers is a continuous negotiation process during the learning and after graduation. The findings are presented according to the interview timeline. The first section describes the repositioning process during learning in ITP based on data collected from interviews in 2017. The second section describes the repositioning process after graduation and return to school. The third section describes contradictory positioning that indicates tensions and dilemmas. Both sections two and three are based on participants’ narratives from interviews conducted in 2019.

In the first section, the shift of participants’ teacher identity to be more inclusive is constructed through different positions associated with inclusivity. During learning, participants were exposed to Finnish inclusive education in this context. They also experienced how inclusive education is implemented on their study programme. Towards the end of their learning, participants described various practices that indicate a more inclusive stance. Participants also described their new understanding of inclusivity in relation to the context and community where they work and where their students come from.

In the second and the third sections, their new teacher identity is further negotiated through repositioning in their post-graduation narratives. Participants’ narratives described efforts to implement some inclusive practices in their schools and classrooms after they graduated and returned to Indonesia. They also position themselves as more inclusive teachers. Their descriptions also contain responses from their students and colleagues to this positioning that described a dialogical process of reconstructing teacher identity to be inclusive teachers.

### 4.1 Participants’ repositioning during learning reflecting the construction of inclusive teacher identity

During learning, participants used diverse positions related to the process of constructing identity as inclusive teachers in their narratives about this period. Participants gained these positions when they interacted with Finnish teacher educators, observed Finnish teachers and reflected on their own positioning in the local context. Participants felt that they were positioned differently when the teacher educators from Finland taught and interacted with them. They also observed how the teacher educators and Finnish teachers in schools positioned themselves in their classrooms or when discussing their teaching experiences with the participants. Participants reported that Finnish teachers were ‘calm’, ‘accept students’ diverse needs', ‘facilitative’, and ‘collaborative’. Participants also described the Finnish teacher educators and Finnish schoolteachers as ‘role models’ in their narratives. Consequently, the participants also tried to emulate these positions themselves in their narratives and even stated that they considered themselves to be role models for their colleagues on their return to school (see Table 2).

**Table 2:** Participants’ descriptions of learning in ITP and Positioning.

Quotes	Position of Finnish Teachers	Positioning of self
” … I want to be a teacher who is psychologically mature, like these Finnish teachers. [I want] to be more consistent and give my best consistently, and to be more positive” (Alya, 2017)	Finnish teachers as role models for maturity	A teacher who wants to emulate maturity by being consistent and positive
“In the learning process, what I am really impressed with is the non-competitive nature of the process. Our teacher [Finnish teacher educator] said ‘if you can all get 5 [the highest marks], no one has to be the best and others lowest’. They teach their students not to compete with each other but to be better than who they were … I used to rank my students and see (mimicking voice) ‘these students are the best and those are the lowest’. Now I think if they can all be the best, why not?" (Fitri, 2017)	Finnish teachers as role models for non-competitive learning	A teacher who tried to avoid labelling students and encourage them to do their best
“We learn how to respect diversity on this programme … we visited the school to observe inclusive education implementation, and I saw how they respect and accommodate diversity in their school … it is important not only to know in theory about inclusive education but to implement it, starting by respecting diversity among ourselves [the group cohort] and help each other … I hope we will implement this in our schools later” (Sinta, 2017)	Finnish teachers respect and accommodate their students' diversity	A teacher who will respect and accept students' diversity and support for each other

In Table 2, the excerpts describe different positioning that participants ascribe to Finnish teachers and positioning that they themselves eventually used in their narratives. In these statements, participants describe a quality that they assume to be related to a certain type of practice. For example, in the first segment, Alya describes the quality of Finnish teachers as psychological maturity, and she further positions Finnish teachers as role models for this quality. She also associates this quality with practices of being consistent and being positive. She then repositioned herself to emulate this quality and implement the practices. Other participants also reported a similar process where they are positioning Finnish teachers role models for quality and practices that they want to emulate.

However, participants also reported that Finnish teacher educators and teachers are ‘conventional’, ‘monotonous’, ‘flat’. This may be attributed to different scenarios about teaching and learning that govern the processes of Finnish and Indonesian teachers, specifically their conception of ‘active students’. While studying on the programme, the participants discovered how activity and passivity in the classroom might not be related to physical movement or level of noise, as they previously believed. Instead, they now associate ‘being active’ with the way students engage with the content and immerse themselves in the learning process. Hence in the turning points of some of their narratives, participants mostly contrasted their previous teaching with their new awareness about engagement and teachers' role that they gain during learning.

“The way the Finnish teacher educators and teachers in the classroom teach is ‘traditional’, they mostly use lecturing and stand in front of the class … if they are supervised by our supervisor from the local ministry of education, they would be called ‘traditional’ and ‘boring’ … yet the students seem to be ready to learn, they sit and listen, they are independent … in our place, there are a lot of pressures to be interesting, use games, be noisy, because our students will easily get bored … here, they teach simply, in our place the teacher has to be entertaining” (Angga, 2017)

Participants' descriptions of previous teaching efforts were primarily associated with finding strategies to keep students attentive in class while being (physically) active. They might disregard what the students needed concerning understanding certain content and mastering specific skills. The recent exposure and discovery during learning and observation on the international teacher programme provided participants with alternative conceptions of students being ‘active’ and ‘engaging’ by acknowledging and accepting their needs and then finding ways to stimulate them to take charge of their learning processes in a more inclusive approach. Moreover, this awareness led to the description of their imagination of the future practices where they tried to respond to diverse students' needs and tailored strategies that might facilitate learning. The following table may describe this process:

**Table 3:** Participants' descriptions of previous teaching practices reflecting ‘active learning scenario in Indonesia’.

Quote	Positioning oneself	Positioning others
” Being a teacher is more than just finding a good teaching strategy or method … we have to know our students … We need to form closer relationships, really knowing the students, their problems, their learning challenges” (Santi, 2017)	As a teacher who understands and builds a relationship with the students	Students with diverse needs for support
” Maybe I have been wrong, sometimes we did not see that the students did not want to study this topic, but maybe they wanted to study something else. I used to force my students, they had to be here and study this, they had to follow … I think I need to teach according to their needs … I must be able to see their unique characters. This is something that I really learn here (Yanti, 2017)	A teacher who used to force students then re-oriented her teaching toward students' needs	Students with unique characteristics
” I think [in Indonesia], we are required to make the class lively, but the students do not learn anything, they only want to play games in class, it is as if they want to avoid studying by playing games … sometimes we do not trust our students to learn. That is what I notice here [in Finland] Finnish teachers trust their students to learn and progress no matter how slight, this intentionality and trust is very visible” (Tina, 2017)	Teachers who teach using creative games to attract students' attention	Finnish teachers who trust their students' learning processes Students who avoid studying and like to play games are easily bored

Table 3 describes three participants' reflections on their previous teaching in Indonesia that may reflect teaching practices in this context. These segments also serve as evaluations of their previous teaching based on their understanding of active learning scenarios in this context. They identify some practices, such as building relationships with students, understanding their learning needs and trusting students that are included in their previous practices. There is a shift from an orientation towards specific teaching and learning scenarios (i.e., active learning) to understanding the diversity of students' characteristics and crafting their responses accordingly. There is repositioning from participants’ initial positioning associated with specific scenarios to new positionings associated with awareness of inclusive pedagogy.

However, the participants also adhered to specific individual plots when describing their experiences. The individual plot organised different ways of being inclusive teachers with diverse practical solutions to implement inclusive education according to their storylines and in response to particular classroom situations. The most commonly used positioning across participants was ‘teachers who accept students’. This acceptance may be related to a more inclusive awareness of students' positioning in the teaching and learning process, including awareness of students' diversity as a group and their uniqueness as individuals. Acceptance also led to the repositioning of these students in their imaginary teaching and learning scenario in the future. The following quote represents a plot and associated positions of self and others reflecting the perspective of inclusive education.

**Table 4:** Evolving individual plots reflecting changes in inclusive education understanding.

Quote	Plot	Position
“I was a victim of a disaster and I got a scholarship to go to school [because of this]. When I studied there, I met good teachers, they taught very well and treated us all the same, no favouritism. And it made me want to become teacher” (Maya 2017)	‘there was no favouritism’	Positions self as a student who experienced merit-based treatment Positions previous teachers as good and fair
“I know more about how to teach, I gain knowledge on how in the classroom, we can't assume all students learn the same way, for example we still teach by giving the same problems to all students and teaching the same way, in Finland is not like that … I want to change when I go home … because we need to treat them equally, so this Finnish system made me think [that]” (Maya 2017)	’I know more about how to teach students equally’	Position self as teacher who teach equally using diverse strategies Position students as having diverse needs and different ways of learning

In Table 4, excerpts from Maya occur in different parts of her narratives. These excerpts describe a repositioning process that occurs throughout the plot of ‘there was no favouritism’ and ‘knowing more about teaching equally’. The first excerpts describe her motivation to become a teacher and being treated fairly in class as a pupil. She stated that fairness was a quality in her teacher which she found inspiring. The second excerpts describe her initial assumptions on how students learn. Her initial practice of giving similar treatments may stem from the practice of segregating students with the same capability and was based on her understanding of fairness. Yet, Maya unfairly assumes her students will learn at the same pace and in the same way, then assumes that students who do not keep up the same pace are ‘failing’. However, her exposure during ITP, provided a new understanding about fairness and equality, one where she needs to acknowledge students' diversity in needs and ways of learning.

The process of participants' identity construction becoming more inclusive through diverse repositioning may reflect exposure to the Finnish inclusivity concept, ‘to provide equal opportunities for all students to learn’ [9]. However, while they are reconstructing their teacher identity, they are also reimagining a more inclusive scenario in teaching and learning based on a new understanding of students' diversity in their context. The repositioning, in some cases, is based on individual plots that organize the way participants make meaning from their diverse learning experiences, reflecting on previous practices and describing their future scenarios in teaching and learning.

### 4.2 Participants’ repositioning of themselves and others in the post-graduation phase related to the implementation of inclusive education in the local context

During the post-graduation phase, participants continued their earlier repositioning during learning and presented themselves as different teachers with practices influenced by a new understanding of being a teacher based on their exposure to Finnish education. Furthermore, based on this understanding, participants also endeavoured to tailor and alternate their teaching strategies dynamically to engage diverse students. Using these new positions, participants experimented with new teaching and learning scenarios associated with the inclusive orientation of providing diverse opportunities for students to learn. The others in their scenarios, possibly their students and other teachers, then respond to these new positions. The responses influenced the dynamic of the repositioning loop during this phase. In the following table, participants describe the changes made during the post-graduation phase and in their new positions (see Table 5).

**Table 5:** Participants’ descriptions of changes implemented after graduation.

Positioning	Quotes
Teacher who includes students' perspectives	“ … as teachers, the initiative may not always start from us. Students have their perspective that has to be acknowledged, usually, we only use our perspective, but we can change position once in a while and see how students view their teachers” (Nardi, 2019)
Teacher who shares assessment policy with her students and supports them to be more critical of their assessment process	“… I tried to request [assessment] rubrics from the teachers, especially for practicum exams … I also shared with my students information about their rights to have clarity and transparency in their project assessment. I told them ‘you should ask for the assessment rubric in every project. In my class this is the rubrics and list of indicators’ … some of our teachers are not used to this communication process” (Tina, 2019)
Teacher who tries to understand students and be more open	“Now, I try to build a relationship with my students by trying to enter their world … at the beginning of term, I tried to win them over … and when I teach, I also share some of my personal stories, my experiences … as a result, they are more respectful to me, and I am closer to them” (Fitri, 2019).

Table 5 presents excerpts from participants describing the new positioning that they used in their narratives after their return to school. These positions indicate new beliefs and practices associated with a more inclusive stance. Some of these positions also reflect more student-oriented practices in their teaching and learning. For example, Nardi described his effort to include students' perspectives, Tina described her practices of sharing assessment policy with her students and Fitri described her efforts to understand her students’ world. For some participants, their new positions in the classroom seemed to be accepted by their school community. Students and colleagues alike might expect the fresh graduates to be different, and participants felt obligated to differentiate themselves from their previous and prevailing practices in school. For example, in their repositioning as student-oriented teachers, participants imagined different scenarios of teaching and learning that also reposition their students differently.

However, this repositioning of self also involved repositioning others and might require these ‘others’ to change along with the process. It started from acknowledging the diversity in their students, for example, participants positioning their students as ‘having diverse needs’, ‘being in different developmental phases’, ‘coming from different backgrounds’, ‘having their own perspectives’. This repositioning of their students shows how participants started to view their teaching as a negotiation process, considering new positions for their students and requiring them to orient their teaching dynamically. These new positions for students also indicate participants' acknowledgement and acceptance of their students' diverse conditions forming the point of departure in their teaching.

In addition to providing a different starting point by including their students' respective conditions and situations, the repositioning also provided an alternative outcome for their teaching process. In some participants' narratives, the repositioning of students as ‘independent’, ‘critical thinkers’ and ‘autonomous’ served as an outcome for their teaching that also reflected participants' previous repositioning of themselves while on the programme and their observations of Finnish classrooms. It is related to the previous repositioning loop during their learning phase, where participants were positioned differently by the teacher educators and themselves. This loop was replicated in participants repositioning their students after graduation.

The participants’ narratives include instances where the repositioning of self and others needs to be adjusted, and scenarios imagined during learning might differ when implemented. In this case, adjusting the repositioning of others and revising their initial self-positioning may require participants to inquire about the change process they planned and implemented. Therefore, the implementation of alternative scenarios for teaching and learning is also sustained by the consistent inquiry process. Some positions may support this process as described in the following table (see Table 6).

**Table 6:** Positions and activities in the inquiry process.

Quotes	Position	Inquiry activity
“I was inspired by the Finnish teacher educators in our programme. One of them described how he did daily observation and was a good observer … so I tried to be like that in my classroom … my previous practice on students' assessment results is usually organised in ranks; I rank them from top to bottom. Now I tried to observe how the students respond to my instrument … which problems are too difficult or too easy, which questions may be difficult to understand … I also requested feedback from the students' (Intan, 2019)	Teacher who observes her assessment process	Evaluate assessment instrument and request feedback from students
“[My research] is relevant, I know the philosophical basis [certain strategy] for example. My current role as school leader is a privilege and provides a bigger platform for me to discuss these issues with students and teachers. For example, bullying, I can distinguish bullying behaviour and help others recognise and differentiate these behaviours in schools … what I told them is based on credible theories … my research is very relevant and helps me share this knowledge with others.” (Angga, 2019)	Teacher who conducts and uses research to inform others	Searching and reading scientific literature on education, analyse and interpret data to inform action
“I joined the Social Teachers' Group Discussion in our school. During the meetings, I usually discuss with other teachers about how to use diverse strategies for engaging and assessing students … when needed, I also observe and teach with them in their classrooms” (Sofia, 2019)	Colleague who supports teachers to implement diverse strategies in the classroom	Using discussion forums and team teaching to learn from each other

As seen in Table 6, participants position themselves as ‘inquirers’ into their practices and existing practices in school. This position is acquired from their learning process in the programme and is eventually used continuously after graduation when they conduct the inquiry process in their teaching and check their students' responses. The inquiry process is also supported by their access to more reading materials while learning and their ability to read materials in English that informed their reflective process about their experience. Some participants described how knowing the more theoretical and philosophical background of teaching and learning helped them in their inquiry and caused them to modify their teaching dynamically to respond to students' diverse needs. With more support, these positions may help participants to continue the change process.

Participants' narratives while learning and after graduation indicate new positions associated with inclusive orientation for practices and relationships in the classroom. These positions help participants form ‘meaning in praxis’ [11] in implementing inclusive education in their context while still retaining some effects of their exposure to the Finnish educational context. Furthermore, the repositioning loop in this phase describes participants' negotiation within the Indonesian context that influences how participants envision a more inclusive teaching and learning scenario and the outcome of their teaching. However, this negotiation may not be free from dilemmas and tensions described in more detail in the following section.

### 4.3 Contradictory positioning in participants’ post-graduation narratives due to implementation challenges in a post-conflict and post-disaster context

Our analysis also identifies some narrative turning points after graduation characterised by dilemmas and tensions, which are often accompanied by reports of confusion and anxiety. The participants also described some contradictory practices due to the existing scenario in inclusive education that may influence the implementation of a new model of inclusive education by participants at school. Akkerman and Meijer [3] argue that when teachers face dilemmas and experience tension in their work, it may be helpful to see it as multiple positions representing conflicting and diverse perspectives.

This section describes the dynamics of positioning at participants' narrative turning points. Participants described tensions regarding the acceptance of other-positioning during learning and implemented after graduation. The following excerpt from Jaka may describe how ‘the others’ in his narrative, in this case, his student, responded to his way of repositioning them. In his narrative, the students in Jaka's narratives refuse to be repositioned and contradict his effort to implement inclusive pedagogy in the classroom.

“ … when I visited [Finnish] school, I met the [same subject] teacher there and he showed me his lesson plan. They prepare lesson plans regularly and simply … although they have a lesson plan, they do not force students to follow this plan strictly. Students have a lot of autonomy here [in Finland], teachers support what students want to learn" (Jaka, 2017)

“So that is what I did. I gave students the freedom to choose first what they want to learn. I invited them to speak out. And I observe the difference between our students and students there [in Finland]. Students there [in Finland] were able to express what they want, they are brave enough to say it, our students are not, they are the types who keep it all in" (Jaka, 2019)

In the first excerpt, while studying on the International Teacher Programme, Jaka repositions himself as someone who, in light of the new teaching and learning scenario observed in Finland, will further promote his students' interests and encourage them to voice their wishes. In the second excerpt, after graduation, he describes his experiment based on this scenario with his students in the Indonesian context. According to his understanding of this approach, he lets his students select what topics or skills they wish to learn and facilitates this in his class. When Jaka repositioned himself, he also unintentionally repositioned his students according to what he had observed in Finnish classes, where students were facilitated and enabled to express their topical interests. However, he found that teaching and learning scenarios in the local context made his students reluctant to voice their ideas openly. This situation inhibited Jaka's effort to include and engage them based on the new scenario. His students were not used to being positioned as the ones to take the initiative in the learning process.

Jaka's narrative indicates his repositioning himself as a teacher who will encourage his pupils to assume responsibility for their learning process. However, the process is not straightforward due to the pre-existing teaching and learning scenarios prevalent in Aceh as a post-conflict and post-disaster context. Specifically in relation to the impact of conflict over the past 30 years, Cardozo & Shah [14] describe how the underlying teaching and learning scenario in Aceh remains unchanged after the rehabilitation process, one that is based on rote learning and scant stimulation for critical thinking. This teaching and learning scenario most probably is the one that Jaka's pupils are used to, this type of scenario asks little of their initiatives and does not encourage his pupils to assess and articulate their own learning needs in their prior learning. As a result, Jaka's invitation for them to assume responsibility for their own learning process may be hampered.

The participants’ post-graduation narratives also include tensions and dilemmas concerning existing scenarios that reflect institutional conditions, such as school accountability mechanisms in the local context. When participants position themselves as inclusive teachers based on their new understanding and implement inclusive teaching according to this understanding, they still need to ensure that their students perform according to the accountability standard stipulated in the Indonesian curriculum. The following excerpts may illustrate these contradictions.

“There was discussion in our meetings about being an inclusive school and achieving minimum competence grades (Ketuntasan Kompetensi Minimum, KKM) … there were questions from the ITP graduates about how inclusive we are in the process [of achieving this grade]? … do we establish different minimum competence for students with special needs? … there is concern that we adhere to formal regulations, so all our students achieved the minimum grade without really providing inclusive learning in the process … we have to understand that academically our students are weak” (Fitri, 2019)

“Some of our teachers understand that students needs various assessments, we cannot use only lecturing and single assessment in teaching ….however when it comes to fulfilling minimum grade requirements, some teachers give students a passing grade but they do not provide diverse assessments in the remedial processes … we are required to ensure our students achieve minimum grade for all subjects, this is hard on our teachers … because our incoming students are not very strong academically” (Tina, 2019)

From these two examples, the tension, dilemma and confusion may come from disagreement about implementing inclusive education during learning while following rigid minimum grades in the assessment process. The excerpts from Fitri and Tina describe one of the contradictions when they position themselves as inclusive teachers who are trying to apply inclusive learning and assessment practices while negotiating the educational accountability requirements in reporting students’ performance.

Moreover, this contradiction is also exacerbated by the reality of Aceh province as a post-conflict and a post-disaster area. These excerpts also describe participants' awareness of the vast pedagogical effort required to implement inclusive education in this area as they understood it from their experience in ITP. Some of their students came from different schooling experiences and were used to different academic demands, described by Fitri and Tina as ‘weak academic background’. As participants try to follow the national curriculum requirements and adhere to minimum grade requirements, their positioning as inclusive educators due to their participation in ITP might stimulate a critical stance toward its implementation. Specifically, when this implementation may disregard reality in the local context and cause schools to implement this regulation as a formality. The assessment then fails to provide insights about students' abilities and does not inform pedagogical decisions in the classroom.

Participants’ new positioning to be inclusive teachers is associated with more efforts to support diversity of learning opportunities and assessment process. However, there are challenges to this implementation due to a rigid accountability system which are based on categorical and segregated ways of managing learning. Additionally, there are complex learning needs due to post-conflict and post-disaster context. For example, the phrases 'weak academically' or 'not very strong academically' represent diverse learning needs due to interrupted and inadequate schooling during conflict and disaster. These quotes seem to represent tensions arising from the pressure to raise the educational standards and accountability of the schools [10]. Bourke further describes that this pressure may lead teachers to use uniform pedagogical and assessment practices, avoid innovation in their assessment and this may have repercussions for pupils.

Participants' identity reconstruction as a more inclusive teacher in this study represented by a pattern of continuous repositioning loop. In addition to reconstructing their identity, their learning in ITP also stimulates reconfiguration of teaching and learning scenarios that may stimulate changes in others. Hence, dilemmas and tensions are also part of participants’ narratives about their learning and actions after graduation. These dilemmas and tensions highlight contradictory positions related to different perspectives on inclusive education in different educational contexts that may challenge the preconditions for implementing a new model of inclusive education in their local context. However, these tensions and dilemmas also point toward possible negotiation directions in the future. In the following section, we discuss the implications of these findings.

## 5 Discussion and conclusion

This study aimed to describe and explore the Indonesian teachers' identity construction process to be inclusive teachers throughout their learning experiences in ITP organised by a Finnish university and two years after they had graduated and returned to school. The findings of this study may support our initial assumption that broadening the inclusive education perspective through this programme is manifest in participants’ repositioning. Furthermore, the findings also underline that repositioning to be more inclusive is associated with narratives about opening access to more learning opportunities by accepting students' diversity.

Based on our analysis of their narratives during these experiences, we can identify the ‘repositioning loop’, a series of repositioning of themselves and others occurring during learning that continues in their school. We also describe how participants' positioning as inclusive teachers based on their exposure in ITP might be negotiated in their storylines as teachers. Additionally, their narratives also indicate tensions and dilemmas where their repositioning loop might be challenged by others and scenarios pre-existing in their context.

Participating in ITP may expose participants to the differing conceptualisation and implementation of inclusive education in another context. In this study, it was the Finnish educational context. The findings support this process as it is reflected in participants' narratives describing negotiation through a series of repositioning using new positions associated with accepting and normalising diversity. For example, the repositioning of Fitri and Sinta may highlight the changes in their understanding about how to manage diversity. Initially, their positioning may indicate common positions for teachers in Indonesian contexts that are based on segregation models for managing diversity ([9,37]). Practices like labelling, ranking and segregating, even as part of inclusive education, may still have a negative impact on its implementation. Specifically, when judgements about categories lead to depriving students of shared learning experiences with their peers. Their narratives describe new positioning after their experience in Finland, as teachers who want to avoid practices like categorising then labelling and teachers who accept students' diverse needs. Participants’ experiences of being positioned differently by Finnish teacher educators during learning also led them eventually to reposition themselves using these new positions. For some participants, these positions also re-orient their positioning toward their students after graduation, creating a continuous repositioning loop throughout the two phases.

Furthermore, the findings also indicate that participants' pedagogical responses come within their life story and episodes [38]. For example, the Maya's plot of ‘there was no favouritism’ and ‘knowing how to teach diverse students equally’ is one example that supports this notion. Hence, these findings also indicate the importance of supporting participants in ITP to construct narratives that will connect the international and novel with the local and familiar as they gain competence in interpreting the needs in their context [34]. The local solutions emerging from this process are diverse and tailored to specific classrooms while negotiated with existing local teaching and learning scenarios. This solution also emerges from the inquiry process supported by new positions associated with this activity, such as observer, reader and collaborative partner.

Earlier research on the impact of ITP hinted at the possible longitudinal and significant impacts of participation in ITP on teachers' identities with its dynamics and complexities. The literature describes this experience as a turning point in their teacher identity trajectory [26] and part of the mobility inherent in all teachers' journeys [5]. Trent [41] also highlights diverse trajectories that student teachers have to negotiate while participating in ITP. The findings in the study support the assumption of ITP participants as a major turning point in identity construction. Specifically, the findings highlight participants’ longitudinal trajectory of being an inclusive teacher even when facing tensions and dilemmas, and this exposure helps them formulate an inclusive stance.

Additionally, the findings also indicate that the questions of permanence reflect the complex and contested nature of turning points. As evidenced by the contradictions in participants’ narratives, the dynamics of the turning points is far from individualised, unified, and continuous. Instead, turning points are also characterised by fragmentation and discontinuity, where detours, hiatus and disruptions exist [27]. However, Bruner [12] associates the turning point with a new stance. Therefore, the impact of participating in ITP for some participants in this study may be construed as a turning point to becoming more inclusive although their identities as inclusive teachers will be continuously negotiated.

This may be related to what Rose & Garner [34] describe as the challenge of transferability from international programme to the local classroom and may influence the permanent state of being an inclusive teacher. The continuous repositioning loop in this study indicates a permanent negotiation process that may continue after they graduate and return to school reflecting the continuous and dynamic state of teacher identity construction [3].

Furthermore, participants' repositioning in this study also has the potential to stimulate a critical view on existing teaching and learning scenarios in their local context. For example, participants' narratives about their previous teaching practices came into question when they observed Finnish classrooms. The active learning scenario that participants understood prior to studying on the international teacher programme positions them to focus on teaching strategy yet overlook students' needs, their relationship with students and trust in them (e.g., Ref. [4]). Additionally, participants also described excessive reliance on specific methods and strategies, such as games, to keep students active although not necessarily engaged. Their awareness of students’ diversity and their inclusive approach may alter the previous positions of teachers and students in these prevailing scenarios and enable them to be more authentic in responding to their students.

Moreover, earlier studies in the implementation of inclusive education in Indonesia described the following factors: the segregated context that challenges the integration of inclusive education into regular schools, the way inclusive as a term may be interpreted within this context and how it influences teachers' perceptions and beliefs about inclusive education ([6,36,39]). The findings from this study describe how these factors were reflected in participants' initial positioning. Furthermore, these factors also feature in the tensions and dilemmas that occur when participants return to school and adopt a critical stance towards school policies. More specifically, their inclusive stance stimulates questions about accountability systems associated with categorisation and narrows the pedagogical responses of teachers in this region. By tracing the specific positioning of inclusive teachers that participants used in their narratives after graduation, the findings provide initial directions as to how inclusivity can play a significant role in improving education in post-conflict and post-disaster contexts. The teacher's inclusive stance based on global citizenry perspective is even more relevant in this process [28].

The tensions and dilemmas also indicate that the participants in this study may have experienced what Forlin [19] described as epistemological dissonance, specifically when they are ‘transposing a new paradigm onto the long-standing traditional educational system that is reluctant to change its basic structure’ (p. 250). For example, Fitri and Tina describe their efforts to implement practices based on a new inclusive perspective within their school's existing school accountability process. The dissonance occurred as they used new positioning associated with a new understanding of inclusive education that stimulated a critical stance. This may need to be explored further by considering the other perspectives in the school community.

Future research may therefore benefit from more data from other school community members to provide additional perspectives about how inclusive education implementation is received and how it may influence the school's teaching and learning process. This approach also opens up the possibility to explore power dynamics in this context. The study has a limitation in analysing participants' internal interpretation rather than the external interactions that may provide different insights. Furthermore, this interpretation is also based on a theoretical perspective and the researchers' understanding of the context. Therefore, there is a possibility that the interpretation of the findings does not fully represent the participants' points of view. Additionally, not all participants volunteered to be respondents in this study. This may also limit the representation of narratives about this programme.

Exposing teachers or student teachers to diversity through ITP or other similar efforts could be considered a significant step towards training more inclusive teachers. Specifically, exposure to different conceptualisations and implementations of inclusive education in another context could be carried further by supporting participants in crafting individual, unique and tailored pedagogical responses. This process underlines the diversity of practices in implementing inclusive education based on equal learning opportunities for all students.

## Author contribution statement

Satia Zen responsible for formulating study's conception and design, data collection, data analysis and interpretation of results, and writing of the manuscripts.

Eero Ropo, Professor and Päivi Kupila, PhD contributed to the study conception and design, data analysis and interpretation of the results, and providing comments on the manuscripts.

## Data availability statement

The data that has been used is confidential.

## Additional information

No additional information is available for this paper.

## Declaration of competing interest

The authors declare that they have no known competing financial interests or personal relationships that could have appeared to influence the work reported in this paper.
